# Effects of Sleepwear Incorporating a DPV576 Functional Polyester Fabric on Wearable ECG-Derived Sleep Consolidation: A Randomized Two-Period Crossover Study Under Free-Living Conditions

**DOI:** 10.3390/s26072247

**Published:** 2026-04-05

**Authors:** Hideki Katano, Masaaki Sugita, Shinichi Tokuno, Yumi Nomura, Naoya Nishino, Masakazu Higuchi, Yusuke Iwai, Yuki Matsuki, Pengyu Deng, Seiji Nishino

**Affiliations:** 1 Faculty of Sport Science, Nippon Sport Science University, Tokyo 158-8508, Japan; katano@recovery.or.jp (H.K.);; 2 Graduate School of Health Innovation, Kanagawa University of Human Services, Yokosuka 238-8522, Japan; 3 Faculty of Creative Engineering, Chiba Institute of Technology, Narashino 275-0016, Japan; 4 Stanford Medicine, Stanford University, Stanford, CA 94304, USA; 5 Faculty of Nursing Department of Nursing, Komazawa Women’s University, Inagi 206-8511, Japan; 6 Graduate School of Health and Sports Science, Juntendo University, Chiba 270-1695, Japan

**Keywords:** functional textile, sleepwear, wearable ECG, actigraphy, sleep efficiency, wake after sleep onset, crossover trial, thermoregulation

## Abstract

**Highlights:**

**What are the main findings?**

- DPV576 sleepwear improved sleep consolidation vs. control (sleep efficiency ↑; awakenings and WASO ↓).
- The condition effects were detectable using wearable ECG-derived sleep metrics with manual verification.

**What are the implications of the main findings?**

- Functional textile sleepwear may offer a low-burden, nonpharmacological strategy to enhance sleep efficiency in free-living settings.
- Wearable ECG endpoints combined with manual checks provide a practical evaluation framework for future registered trials of textile interventions.

**Abstract:**

Sleep quality is essential for maintaining physical health and psychological resilience. Because sleepwear remains in direct contact with the skin throughout the night, it may affect thermoregulation and comfort and, thereby, influence sleep. This randomized two-period, two-sequence crossover study investigated whether sleepwear infused with nanodiamond and nanoplatinum particles (DPV576) could improve sleep quality and promote fatigue recovery under free-living conditions. Fourteen healthy men (23.9 ± 1.7 years) wore DPV576 sleepwear and visually indistinguishable standard polyester sleepwear for one week each, separated by a one-week washout. Sleep was assessed using a wearable ECG-based actigraphy device; trained researchers additionally performed manual rescoring to verify automated outputs, including independent determination of sleep onset latency. Subjective sleep was assessed daily using the Sleep Quality Index of Daily Sleep and a visual analog scale; exploratory outcomes included voice-derived biomarkers and pre-/post-sleep grip strength. In manual rescoring, DPV576 was associated with higher sleep efficiency (93.0 ± 0.9% vs. 89.5 ± 1.5%, *p* < 0.05), fewer awakenings (8.4 ± 1.3 vs. 10.7 ± 1.4, *p* < 0.01), and shorter wake after sleep onset (30.4 ± 4.7 vs. 41.6 ± 6.0 min, *p* < 0.01), whereas total sleep time did not differ significantly (*p* = 0.096). These findings suggest that one-week use of DPV576 sleepwear may improve wearable ECG-derived sleep consolidation in young men, supporting a nonpharmacological wearable strategy to enhance sleep efficiency in everyday settings.

## 1. Introduction

Adequate sleep is essential for sustaining physiological health and cognitive resilience. Chronic sleep deprivation has been linked to various lifestyle-related diseases such as hypertension, diabetes, dyslipidemia, myocardial infarction, and cerebrovascular disorders [1,2]. Chronic sleep deprivation may also compromise immune function, increasing susceptibility to infectious diseases such as influenza and potentially contributing to cancer progression [3]. In Japan, approximately 21.4% of the population reports symptoms of insomnia, such as difficulty initiating or maintaining sleep [4], and with a higher prevalence among adolescents (23.5%) [5]. Given the rise in psychosocial stressors, the proportion of people reporting insomnia is likely to increase, potentially increasing the burden of associated illnesses. Therefore, developing effective sleep-improvement interventions is of significant societal importance. Achieving healthy sleep depends not only on individual behavior but also on optimization of the external sleep environment. To maintain a favorable external sleep environment, measures such as selecting suitable bedding and ensuring a quiet, dark, and thermally comfortable room are recommended. Comfort during sleep enhances sleep quality, which in turn supports optimal physical health [6].

One often overlooked factor is sleepwear, which remains in direct contact with the skin and can affect thermal comfort through its material properties. As a proximal layer, sleepwear contributes to the thermoregulatory microclimate (air temperature and humidity) at the skin–textile–bedding interface [7,8]. Thus, sleepwear can influence heat dissipation patterns around sleep onset. In recent years, functional garments made from sweat-absorbing and heat-retaining fibers have gained popularity, particularly during colder seasons, due to their thermal properties [9]. These materials are designed to regulate moisture transport and insulation, thereby stabilizing perceived warmth. Such garments help regulate the skin’s microclimate, promoting thermal comfort and potentially enhancing sleep quality. Improved sleep, in turn, is known to support cognitive and physical performance [10,11,12,13,14]. However, the direct effects of such garments on sleep quality and post-sleep recovery remain unclear.

This study aimed to evaluate the effects of sleepwear containing nanodiamond-nanoplatinum (DPV576) on objective and subjective sleep quality and recovery-related outcomes in healthy adult men, while exploring downstream functional outcomes as hypothesis-generation endpoints. In a randomized two-period crossover under free-living conditions, we hypothesized that wearing DPV576-infused sleepwear during sleep would improve sleep continuity, particularly sleep efficiency and nocturnal awakenings.

## 2. Materials and Methods

### 2.1. Participants

Fourteen healthy adult men (mean age: 23.9 ± 1.7 years) were recruited for the study. Written informed consent was obtained from all individuals prior to participation. The study was approved by the Ethics Committee of Nippon Sport Science University (Approval No. 019-H083), and all procedures conformed to the Declaration of Helsinki (2013 revision).

### 2.2. Study Design

We conducted a randomized two-period, two-condition crossover trial under free-living conditions. The study was intentionally conducted under free-living conditions to evaluate the intervention in an environment as close as possible to participants’ usual daily lives. During each study period, participants were instructed to maintain their usual daily routines, keep their sleep schedule as consistent as possible, and sleep in their usual home environment each night. They were also asked to maintain similar dietary and caffeine intake across conditions, abstain from alcohol during the testing period, and avoid foods or medications that could affect sleep before bedtime. Participants alternately wore either sleepwear incorporating a DPV576 functional polyester fabric (experimental) or standard polyester sleepwear (control) for one week each, separated by a one-week washout. The order of conditions was randomized using a computer-generated allocation list. The two garment conditions were visually indistinguishable; accordingly, participants were blinded to the condition assignment. Data analysts were likewise blinded to the coded condition labels during the primary analyses. Statistical analyses were performed at the night level; nights that failed device validity criteria were excluded, and only valid paired observations were included in the within-subject comparisons. After the completion of the primary analyses, the condition labels were unblinded for reporting, and treatment contrasts are presented as Δ = DPV576–control.

### 2.3. Interventions (Figure 1A)

DPV576 sleepwear (experimental): short-sleeved shirt and long trousers made of polyester fibers containing nanodiamond (ND) and nanoplatinum (NP), embedded within the fibers; material supplied by Venex Co., Ltd. (Atsugi, Kanagawa, Japan). Control sleepwear consisted of a standard polyester short-sleeved shirt and long trousers without DPV576. The DPV576 and control garments were matched in type and external appearance, so that participants were unable to distinguish between the two conditions.

**Figure 1:**
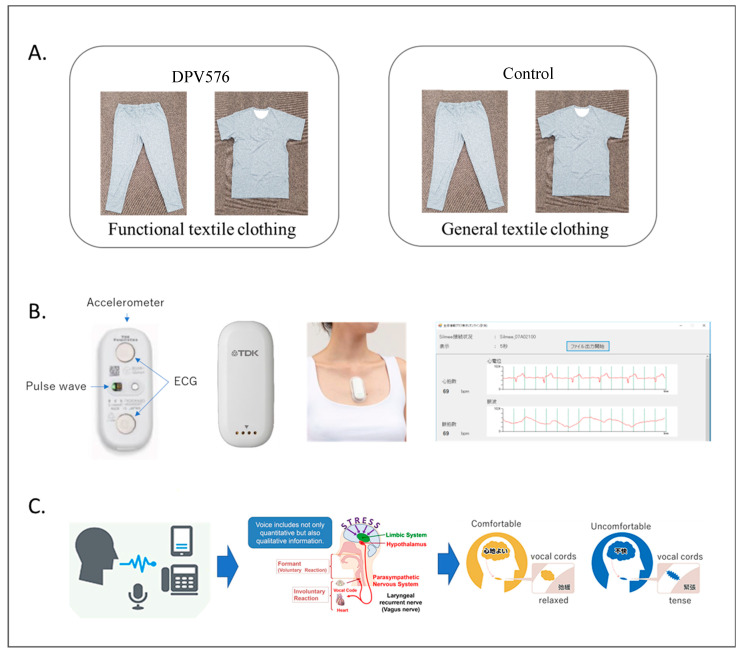
(**A**) Garment conditions: functional textile clothing (DPV576) and control clothing (standard textile); (**B**) Wearable measuring device (Silmee™ Bar type Lite (TK010HM00; TDK Corporation, Tokyo, Japan); Electrocardiogram), (**C**) Voice analysis technology (MIMOSYS: Mind Monitoring System).

### 2.4. Outcomes

#### 2.4.1. Pittsburgh Sleep Quality Index (PSQI)

A validated instrument assessing habitual sleep and potential disturbances over the past month [15,16,17], comprising seven component scores summed to a global score (0–21; higher scores indicate worse sleep); administered at baseline before randomization.

#### 2.4.2. Epworth Sleepiness Scale (ESS)

An 8-item scale assessing daytime sleepiness (total score 0–24; higher scores indicate greater sleepiness); administered at baseline [18].

#### 2.4.3. Post-Randomization Daily Questionnaires

##### Sleep Quality Index for Daily Sleep (SQIDS)

Assesses daily sleep status; seven items scored 0–2 or 0–3 for a maximum of 20 [19], higher indicating poorer sleep; completed each study day.

##### Visual Analog Scale (Sleep VAS)

A 100 mm line scale assessing last night’s sleep quality, current drowsiness, current mood, and current activity [20]; participants marked each item daily.

#### 2.4.4. Measurements

Objective sleep parameters were monitored using a wearable ECG sensor (Silmee Bar Type Lite, TDK Corporation, Tokyo, Japan) attached to the chest with a conductive gel pad (Figure 1B). Variables included total sleep time (TST), sleep efficiency (SE), sleep onset latency (SOL), time in deep sleep, number of awakenings, wake after sleep onset (WASO), body-movement frequency (≥0.05 G), and heart-rate variability (HRV) indices: LF/HF ratio with LF 0.04–0.15 Hz and HF 0.15–0.40 Hz [21,22,23]. Automated sleep–wake staging and SOL identification followed the device’s validated algorithms.

#### 2.4.5. Manual Scoring

Because SOL influences TST and SE, trained researchers independently performed manual scoring to verify automated outputs, including independent determination of SOL. Both automated and manual pipelines were retained for analysis, and the concordance between pipelines was evaluated at the outcome level.

#### 2.4.6. Ancillary Voice-Based Measure

Psychophysiological state was assessed using the MIMOSYS voice-analysis system (University of Tokyo), which computes a “Vitality Index” and “Mental Activity” from standardized reading tasks [24,25,26]. These measures were collected daily post-awakening to index short- and longer-term affective state. (Figure 1C).

#### 2.4.7. Grip Strength

Physical recovery was assessed using a digital grip-strength meter (Grip D, Takei Kikai Kogyo). Three trials per hand were obtained before bedtime and after awakening on each study day, following standardized posture and effort instructions; the mean of three trials per hand was used for analysis.

### 2.5. Statistical Analysis

Data were expressed as mean ± standard error (SE); 95% confidence intervals (CIs) for condition means were calculated as mean ± 1.96 × SE, and 95% CIs for paired contrasts were obtained from the t distribution of paired differences, with Δ defined as DPV576–control. The randomized two-period, two-sequence crossover design was analyzed within subjects during the night level and valid paired nights were included (n = 14). For continuous outcomes (SE, WASO, SOL, TST, grip strength and questionnaires), normality of paired differences was assessed using the Shapiro–Wilk test; when approximately normal, two-sided paired *t*-tests were used, and when non-normal, the Wilcoxon’s signed-rank test was additionally performed on the Δ values to confirm conclusions. HRV (LF/HF) was summarized by quarter of the night (Q1–Q4) from the ECG signal, using a within-subject repeated-measures ANOVA with condition (DPV576 vs. control) as the within factor and period screened as a fixed effect. Period/sequence (carryover) effects were screened; no material effects were detected, and comparisons collapsed across periods. Missing observations were handled using the available-pair analysis without imputation. Analyses were done by a version of the software R (Version 4.x) using the lme4, lmerTest and emmeans packages. Statistical significance was set at *p* < 0.05.

## 3. Results

**Figure 2:**
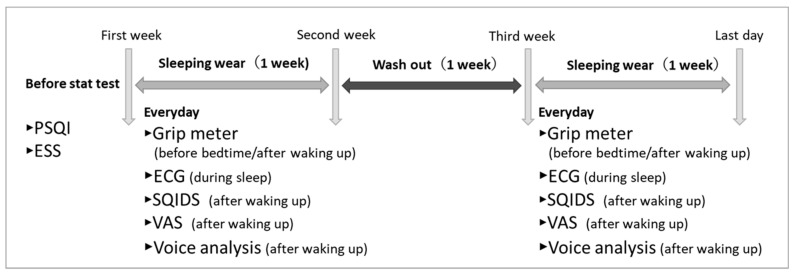
Experimental design (PSQI: Pittsburgh Sleep Quality Index, ESS: Epworth Sleepiness Scale, Grip meter, Grip strength measurement, ECG: Electrocardiogram, SQIDS: Sleep Quality Index for Daily Sleep, VAS: Visual Analog Scale, Voice analysis, Analysis of “Vitality Index” and “Mental Activity”).

**Figure 3:**
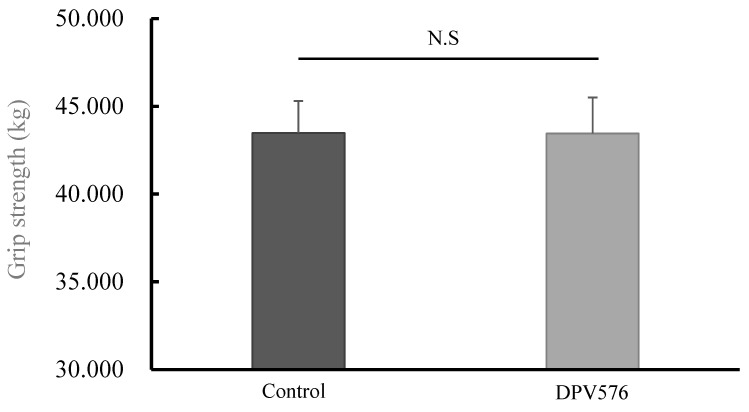
Comparison of mean grip strength over 7 days by condition. n = 14. N.S.: Not significant.

**Figure 4:**
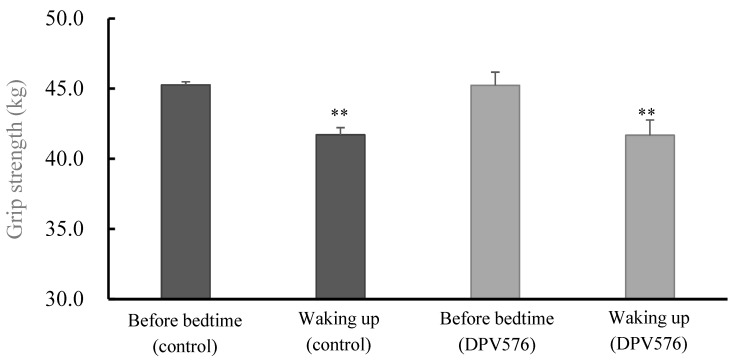
Comparison between before bedtime and after waking up over 7 days by condition. n = 14. ** vs. Before bedtime, *p* < 0.01.

We analyzed within-subject paired observations from the randomized two-period crossover (Δ = DPV576–control). Night-level pairs meeting device-validity criteria were included (n = 14). Main-text tables and figures are as in the original manuscript (Table 1, Table 2, Table 3 and Table 4; Figure 1, Figure 2, Figure 3 and Figure 4).

### 3.1. Baseline Questionnaire Responses

#### 3.1.1. Pittsburgh Sleep Questionnaire (PSQI)

57.1% scored ≥6 (potential sleep disturbance) and 35.8% scored <6; 7.1% scored ≥10, indicating more severe problems (Table 1). These baseline profiles indicate room for improvement, consistent with the physiological direction later observed on actigraphy.

#### 3.1.2. Epworth Sleepiness Scale (ESS)

64.3% reported no excessive daytime sleepiness (0–5), 35.7% mild (6–10), and none severe (≥11) (Table 1).

### 3.2. Questionnaire Survey Results

#### 3.2.1. Sleep Questionnaire (SQIDS)

28.6% improved with DPV576, 28.6% worsened, and 42.8% were unchanged; the mean total score was 4.0 ± 0.4 in both conditions, indicating a negligible between-condition contrast over the brief crossover (Table 1).

#### 3.2.2. Visual Analog Scale (VAS)

For DPV576 vs. control, VAS scores were 37.2 ± 1.2 vs. 40.1 ± 1.4 mm for sleep quality, 39.5 ± 1.4 vs. 38.0 ± 0.8 mm for drowsiness, 32.5 ± 0.8 vs. 33.9 ± 0.6 mm for mood, and 33.5 ± 1.2 vs. 34.1 ± 0.5 mm for activity. No significant differences were observed between conditions (Table 1).

### 3.3. Objective Sleep Measures: Automated Scoring (Silmee)

Table 2 summarizes outcomes obtained by automated scoring (including identification of sleep onset latency). Sleep efficiency (SE) was 92.9% [95% CI 90.5–95.3] with DPV576 than 90.2% [87.8–92.6] with control (Δ = +2.7 percentage points; *p* < 0.05). Awakenings were 8.2 [95% CI 5.8–10.6] vs 10.6 [7.9–13.3] per night (Δ = −2.4; *p* < 0.05), and wake after sleep onset (WASO) was 29.5 min [20.5–38.5] than 41.2 min [29.4–52.9] (Δ = −11.7 min; *p* < 0.05). Total sleep time was 392 min [95% CI 364.6–419.4] vs 419 min [95% CI 385.7–452.3] (*p* = 0.053). Sleep onset latency, deep sleep duration, and body movement frequency showed no between-conditions differences.

### 3.4. Objective Sleep Measures: Manual Scoring

Table 3 summarizes manually rescored outcomes (including independent identification of sleep onset latency). SE remained significantly higher with DPV576 (93.0% [95% CI 91.2–94.8]) vs. control 89.5% [86.6–92.4] (*p* < 0.05), with fewer awakenings (8.4 [5.9–10.9]) vs. 10.8 [8.1–13.5]; *p* < 0.05) and shorter WASO (30.4 min [21.2–39.6) vs. 41.6 min [29.8–53.4]; *p* < 0.05)). Total sleep time did not differ significantly (*p* = 0.096).

### 3.5. Comparing Automated and Manual Scoring

Apart from expected differences in absolute sleep onset latency across pipelines (automated 20.4 ± 4.6 min; manual 14.3 ± 2.3 min), both pipelines yielded concordant outcomes: higher SE, fewer awakenings, and shorter WASO with DPV576 vs. control (Table 2 and Table 3). Using the paired within-subject analysis, the condition effects were SE Δ = +3.1 with percentage points [95% CI −0.4 to +6.6] and SOL Δ = −3.9 min [95% CI −11.4 to +3.7], mirroring the direction shown in Table 2 and Table 3.

#### Autonomic Indices (HRV)

The LF/HF ratio derived from the ECG signal was summarized by quarter of the night (Q1–Q4) in Table 4. Compared with control, the DPV 576 condition showed similar LF/HF values in Q1–Q3 and a higher value in Q4 (2.09 ± 0.18 vs. 1.81 ± 0.18), yielding a nominal unadjusted difference at Q4 (*p* = 0.016). Given the pilot sample and multiple quarter-specific comparisons, this finding should be interpreted cautiously. Night-averaged LF/HF did not differ between conditions.

### 3.6. Grip Strength

Figure 3 and Figure 4 show mean grip strength across the 7-day window. Grip strength decreased from bedtime to wake-up in both conditions (control: 45.3 ± 0.2 to 41.7 ± 0.5 kg; DPV576: 45.2 ± 0.9 to 41.7 ± 1.1 kg; *p* < 0.05), with no significant between-condition difference.

## 4. Discussion

In this pilot-randomized crossover study, one-week use of functional polyester sleepwear, incorporating nanoplatinum and nanodiamond (DPV576), was associated with improved objective sleep quality, as reflected by higher sleep efficiency, fewer awakenings, and shorter wake after sleep onset (WASO). These findings are consistent with a possible role of sleepwear-related thermal microclimate in sleep health [27,28].

These findings are broadly consistent with previous studies suggesting that sleep-related clothing interventions may influence objective sleep parameters, potentially through thermal mechanisms. Foot warming with bed socks in a cool environment was reported to improve sleep onset latency, sleep efficiency, total sleep time, and nocturnal awakenings, although subjective ratings did not differ significantly from control condition [29]. Similarly, a randomized crossover study, comparing sleepwear fiber types under warm conditions, found modest but significant benefits of certain materials for sleep onset and sleep fragmentation, particularly in older adults and poorer sleepers [30]. A recent systematic review further concluded that sleepwear and bedding fiber types may affect sleep quality, although the available evidence remains heterogeneous [31]. In this context, the present study extends the existing literature by showing improved sleep continuity with functional sleepwear under free-living conditions, using wearable ECG-derived out-comes with manual verification.

The preliminary survey using the PSQI revealed that more than half of the participants exhibited poor sleep quality, consistent with previous studies of young adult populations [32,33,34]. ESS indicated that none of the participants exhibited excessive daytime sleepiness or symptoms indicative of sleep apnea. After the experiment began, the SQIDS questionnaire [35] and a daily sleep VAS [36] were administered as subjective measures of sleep quality. After randomization, neither SQIDS scores nor daily sleep VAS ratings differed significantly between conditions, despite improvements in objective sleep continuity measures. This discrepancy is not uncommon, as subjective and objective sleep assessments do not always closely correspond and may reflect partly different aspects of sleep [37,38]. In the present study, the participants were healthy young men, and the intervention period was short, so subjective daily questionnaires may have been less sensitive to modest changes in sleep continuity. Accordingly, the present findings suggest short-term objective benefits, but the extent to which these changes translate into perceptible or clinically meaningful subjective improvement, remains to be determined in longer studies and in populations with greater baseline sleep complaints. Sleep onset latency, deep sleep duration, body movement frequency and LF/HF did not differ significantly between conditions. Given the pilot sample size and the number of outcomes assessed, these findings should be interpreted cautiously and regarded as secondary or exploratory rather than confirmatory. This pattern is consistent with previous research suggesting that objective improvements in sleep do not always translate into detectable changes in subjective sleep perception, particularly in short-term interventions. For the ancillary voice-based measures (MIMOSYS), the Vitality Index and Mental Activity were directionally higher in the test garment condition, but these differences were not statistically significant. Morning grip strength declined in both conditions, consistent with previous finding [39] and with known diurnal variation and transient sleep inertia, with no between condition differences. These ancillary findings should likewise be interpreted cautiously and are better regarded as hypothesis-generating than confirmatory. This post-sleep reduction likely reflects normal physiological processes, including muscle relaxation during sleep, reduced core body temperature upon awakening, and transient delays in neuromuscular activation [40].

Sleep regulation reflects the interaction of homeostatic and circadian processes coupled with thermoregulation, with the preoptic/anterior hypothalamus orchestrating heat-loss behaviors and cutaneous vasodilation [41,42]. The homeostatic process refers to the buildup of sleep pressure during wakefulness, which decreases during nighttime sleep [43]. The circadian rhythm, regulated by the suprachiasmatic nucleus, helps align sleep timing with environmental cues such as light and body-temperature rhythms [8,44,45]. Moreover, thermogenesis pathways are located in the anterior hypothalamus and are closely linked to circadian regulation [46]. Previous findings suggested that increase in skin temperature helps determine the timing of sleep; as sleep onset approaches, peripheral blood vessels dilate, skin blood flow increases, and heat dissipation rises, resulting in an increase in distal (limb) skin temperature accompanied by a decrease in core body temperature [47]. Peripheral vasodilation, particularly in the fingers, facilitates heat dissipation in the hours preceding sleep onset and into early sleep [48]. High-temperature bathing immediately before bedtime blunts the requisite decline in core body temperature, thereby hindering sleep initiation and degrading sleep quality. Conversely, wearing a thermal suit during sleep that raises skin temperature by approximately 0.4 °C has been reported to reduce nocturnal awakenings [28]. Together, these observations support the plausibility of a thermophysiological link between clothing microclimate and sleep continuity [49,50]. However, because skin surface temperature and heat dissipation were not measured in the present study, this interpretation, therefore, remains speculative.

The FIR-emitting properties of DPV576 fabrics may provide one possible physiological context for the present findings, as FIR-related interventions have been associated with peripheral circulation and thermal responses in previous studies [49,51]. However, the present study did not directly include the measuring of thermal, endocrine, or targeted autonomic variables. Therefore, these pathways should be regarded as possible background mechanisms rather than demonstrated explanations for the current results. Likewise, because neither next-morning grip strength nor total sleep time differed between conditions, any broader recovery-related interpretation should be considered hypothesis-generating rather than confirmatory. Targeted, time-of-day-controlled assessments of performance and fatigue will be needed to evaluate this translational possibility. Within the limits of the present data, DPV576-infused sleepwear may represent a novel, noninvasive approach for improving sleep continuity in everyday settings, while the underlying physiological mechanisms remain to be clarified in future studies with direct measurement.

### Limitations

Several limitations should be acknowledged in this pilot study. First, the sample size was small (n = 14), and all participants were healthy young men. Therefore, the generalizability of the findings to women and other age groups remains to be established. Second, each intervention period lasted only one week. Accordingly, the present study addresses short-term effects on sleep continuity, but cannot determine whether these effects are sustained over longer periods or accompanied by longer-term physiological adaptation [52]. Third, although participants were instructed to maintain consistent sleep routines and home sleeping conditions, the trial was conducted under free-living conditions. As a result, bedroom temperature and humidity were not directly measured, and behavioral factors such as diet and caffeine intake could not be controlled as strictly as in a laboratory setting [53,54]. Fourth, the study did not directly assess the physiological variables needed to test the proposed thermoregulatory or autonomic mechanisms, such as skin surface temperature, heat dissipation, or targeted autonomic measurements during sleep. Therefore, any mechanistic interpretation should be considered hypothesis-generating rather than confirmatory [55]. Direct physiological measurements will be needed to determine whether DPV576 influences sleep continuity through thermal and/or autonomic pathways.

## 5. Conclusions

In a two-period randomized crossover trial conducted under free-living conditions, one-week use of DPV576-infused sleepwear was associated with improved objective sleep continuity, particularly higher sleep efficiency, fewer awakenings, and shorter WASO, in healthy young men. These findings suggest that DPV576-infused sleepwear may have potential as a nonpharmacological approach to improving sleep continuity.

## Figures and Tables

**Table 1:** Questionnaires: baseline PSQI/ESS; SQIDS and VAS by condition.

(A) Baseline Questionnaires			
PSQI category	n	%	
≥6 (potential disturbance)	8	57.1	
<6 (no disturbance)	5	35.8	
≥10 (more severe)	1	7.1	
ESS category			
0–5 (no EDS)	9	64.3	
6–10 (mild EDS)	5	35.7	
≥11 (severe EDS)	0	0.0	
**(B) Condition-Level Questionnaires During the Trial**			
**Scale**	**DPV576**	**Control**	**Condition** ***p*** **-Value**
SQIDS (total)	4.0 ± 0.4	4.0 ± 0.4	0.536
VAS—sleep quality (mm)	37.2 ± 1.2	40.1 ± 1.4	0.186
VAS—drowsiness (mm)	39.5 ± 1.4	38.0 ± 0.8	0.776
VAS—mood (mm)	32.5 ± 0.8	33.9 ± 0.6	0.314
VAS—activity (mm)	33.5 ± 1.2	34.1 ± 0.5	0.768

Notes: Mean ± SE. VAS scaled 0–100 mm. Between-condition comparisons used within-subject paired tests; PSQI: Pittsburgh Sleep Quality Index; ESS: Epworth Sleepiness Scale; SQIDS: Sleep Quality Index of Daily Sleep. n = 14.

**Table 2:** Analysis of results from automated scoring of sleep onset latency by wearable measuring instrument.

	DPV576 (n = 14)			Control (n = 14)			Difference			*p*-Value	
	Mean		SE	Mean		SE	Mean		SE		
Sleep Time (min)	392	±	14	419	±	17	−26	±	12	0.053	
Sleep Efficiency (%) ^1^	92.9	±	1.0	90.2	±	1.2	3.0	±	0.7	0.002	**
Sleep Onset Latency (min)	20.4	±	4.6	21.9	±	3.8	−1.4	±	5.9	0.814	
Deep Sleep Time (min)	68.5	±	6.3	68.0	±	7.0	0.5	±	6.0	0.938	
Number of Awakenings	8.2	±	1.2	10.6	±	1.4	−2.4	±	0.6	0.002	**
Time Awake (min)	29.5	±	4.5	41.2	±	6.0	−11.7	±	3.2	0.003	**
Body Movement Frequency ^2^	0.1	±	0.0	0.1	±	0.0	0.0	±	0.0	0.283	

Notes: Mean ± SE; ^1^. Sleep Efficiency: Asleep time (the amount of time spent asleep (in minutes))/Sleep time (the total amount of time in bed (in minutes)) × 100; ^2^. Body Movement Frequency: Average amount of body movement (number of movements of 0.05 G or more per minute); n = 14. ** *p* < 0.01.

**Table 3:** Analysis of results from the researcher’s manual scoring for the identification of Sleep Onset Latency by Wearable Measuring Instrument.

	DPV576			Control			Difference			*p*-Value	
	Mean		SE	Mean		SE	Mean		SE		
Sleep Time (min)	399	±	16	0.42	±	17	−23	±	12	0.096	
Sleep Efficiency (%) ^1^	93.0	±	0.9	89.5	±	1.5	3.5	±	1.2	0.015	*
Sleep Onset Latency (min)	14.3	±	2.3	20.5	±	4.1	−6.2	±	4.6	0.202	
Deep Sleep Time (min)	69.0	±	6.3	68.7	±	6.9	0.4	±	6.3	0.954	
Number of Awakenings	8.4	±	1.3	10.7	±	1.4	−2.3	±	0.6	0.001	**
Time Awake (min)	30.4	±	4.7	41.6	±	6.0	−11.2	±	3.4	0.006	**
Body Movement Frequency ^2^	0.1	±	0.0	0.1	±	0.0	0.0	±	0.0	0.072	

Notes: Mean ± SE; ^1^. Sleep efficiency: Asleep time (the amount of time spent asleep (in minutes))/Sleep time (the total amount of time in bed (in minutes)) * 100; ^2^. Body movement frequency: Average amount of body movement (number of movements of 0.05 G or more per minute). n = 14. * *p* < 0.05, ** *p* < 0.01.

**Table 4:** Heart-rate variability (LF/HF) by quarter of the night (Q1–Q4).

	1/4			2/4			3/4			4/4				Type	Time	Type * Time
Control (dimensionless)	1.79	±	0.18	1.64	±	0.18	1.77	±	0.18	1.81	±	0.18	F-value	0.41	0.91	0.50
DPV576 (dimensionless)	1.84	±	0.18	1.71	±	0.18	1.81	±	0.18	2.09	±	0.18	*p*-value	0.3049	0.0164 *	0.3049

Notes: Mean ± SE; LF 0.04–0.15 Hz; HF 0.15–0.40 Hz; LF/HF dimensionless. n = 14. * *p* < 0.05.

## Data Availability

The datasets generated during the current study will be made available by the corresponding author on reasonable request due to ethical and privacy restrictions related to human participant data.
